# Report of the Organization of the Dental Association of Allegheny County, Pa

**Published:** 1853-07

**Authors:** 


### *Report of the Organization of the Dental Association of Allegheny County, Pa.*

In compliance with an invitation of several members of the dental profession, an assembly, composed of a large majority of the dentists of Allegheny county, met at the house of a member of the profession, in Fourth street, Pittsburg, at seven o'clock, P. M., on the 2d of Oct., 1852.

On motion, it was resolved, that J. Scott, of Pittsburg, preside at the first meeting as Chairman, and M. Depuy act as Secretary.

The meeting was then called to order by the President. By request, the letter of invitation calling the meeting was read, and the objects of it stated by the Chair.

A general discussion ensued, on the expediency of making an effort to form an organization for the improvement and elevation of the Dental profession in this vicinity.

On motion, it was then resolved, that a committee of three be elected to draft a constitution and by-laws, to be submitted for consideration at a future meeting.

The following committee was then elected by ballot:—W. M. Wright, W. F. Fundenberg and Wm. Bachop.

After some further discussion, on the best method of conducting an association, the meeting adjourned until Oct. 16th, at half-past 7 o'clock, P. M., to meet at the same place.

Oct. 16, 7½ P. M.—An assemblage of the Dental profession convened, according to adjournment.

The meeting was called to order by the Chair, when the committee elected to draft a constitution and by-laws, presented their report.

On motion, it was resolved to take up the articles of the constitution and by-laws separately, and, after canvassing their merits, adopt, amend, or reject them, by vote.

The constitution and by-laws, after being amended in a few particulars, were, on motion, declared to be adopted as the constitution of the "Dental Association of Allegheny County, Pa."

On motion, it was then resolved, that the meeting go into an election for officers, according to the provisions of the constitution, for the ensuing year.

The following gentlemen were then elected, by ballot:—Dr. W. M. Wright, President; John Scott, Vice President; M. Depuy, Secretary; Robert Vandevort, Treasurer.

An Abstract of the Constitution and By-Laws.—The name of this society shall be, "*The Dental Association of Allegheny County, Pa.*"

Application for membership must be made in writing, and be acted on at the next ensuing meeting. Three-fourths of the ballots shall be necessary for choice.

The officers of the Society shall be, a President, Vice President, Secretary, and a Treasurer.

The duties of the above are the same as are usually incident to the same in other societies.

Duties of Members.—The members are enrolled in alphabetical order. Each member, in the order in which his name occurs, reads before the Society, an essay on some subject connected with dentistry, so that there shall be at least one essay read at each stated meeting of the Society.

The stated meetings of the Society are to be held on the first Saturday of each month, at 7 o'clock, P. M.

The stated meeting in October, shall be considered the Annual Meeting.

No member of this Association shall patent any instrument, or invention, that might be of service to other members of the dental profession.

No member of this Association shall take a student for a shorter term than two years.

The above abstracts presents some of the leading features of the constitution and by-laws of the Society. The meetings, thus far, have been well attended and interesting, and it remains to be seen if it will fulfill the expectations of its founders, and allay animosities and promote a feeling of good-fellowship among the members of our profession in this region.—*Dental News Letter.*

